# Enhanced Skin Permeation and Pigmentation Reduction Effects of a Novel Tranexamic Acid‐Mandelic Acid Ion‐Pairing Complex

**DOI:** 10.1111/srt.70222

**Published:** 2025-09-11

**Authors:** Hyungjoon Jeon, Nojin Park, Jong Gu Won, Yong Won Shin, Jiwon Choi, Kyoungin Min, Eunjung Choi, Chang Kyu Kim, Sang‐Wook Park, Nam Seo Son

**Affiliations:** ^1^ LG Household & Health Care (LG H&H), LG Science Park Seoul Republic of Korea

**Keywords:** inflammation, ion‐pairing, mandelic acid, skin permeation, skin pigmentation, tranexamic acid

## Abstract

**Purpose:**

This study investigated the enhanced skin permeation and pigmentation reduction effects of an ion‐pair complex formed between tranexamic acid (TXA) and mandelic acid (MA). The TXA‐MA complex demonstrated superior skin permeability and greater inhibition of cytokine expression compared to TXA alone, ultimately proving more effective in reducing skin pigmentation.

**Methods:**

After spectroscopic analysis of the TXA‐MA ion‐pairing complex (TXA‐MA complex) structure, an in vitro skin permeation study was conducted using a Franz cell system with porcine skin. Additionally, the effect of the TXA‐MA complex on UVB (ultraviolet B)‐induced expression changes of inflammation‐related genes (IL‐1α, IL‐6, IL‐8, COX2) was evaluated using a human epidermal keratinocyte cell lines (HaCaT) cell model. Finally, an in vivo human study was performed to analyze the efficacy of TXA‐MA in reducing actual skin pigmentation.

**Results:**

The formation of the TXA‐MA complex was confirmed through zeta‐potential measurements, ^1^H NMR study, and Fourier‐transform infrared spectroscopy (FT‐IR) spectroscopy. Skin permeation studies using porcine skin showed enhanced permeability of the TXA‐MA complex compared to TXA at equivalent concentrations. In the HaCaT cell model, the TXA‐MA complex exhibited greater inhibition of inflammatory markers IL‐1α, IL‐6, IL‐8, and COX‐2 expression than TXA alone. Finally, a 4‐week human clinical study using ANTERA 3D imaging demonstrated that the TXA‐MA complex was significantly more effective than TXA alone in reducing skin pigmentation.

**Conclusion:**

This study successfully formed a TXA‐MA complex. Compared to TXA, the TXA‐MA complex showed superior effects in skin permeability, inhibition of inflammatory marker expression, and actual skin pigmentation reduction. These results suggest that the TXA‐MA complex holds greater potential as an effective cosmetic ingredient for pigmentation improvement than TXA alone.

## 1 Introduction

Skin pigmentation issues, including hyperpigmentation and uneven skin tone, are significantly influenced by inflammatory processes in the skin. Inflammation triggers the release of pro‐inflammatory cytokines (e.g., IL‐1α, IL‐6, TNF‐α) and mediators (e.g., α‐MSH, SCF) that stimulate melanocytes through PAR‐2 and NF‐κB pathways, leading to increased melanin synthesis and deposition [1, 2]. Ultraviolet B (UVB) radiation amplifies this process by upregulating tyrosinase activity and MITF expression via ROS‐dependent ERK/MAPK signaling, while simultaneously inducing keratinocytes to secrete IL‐1β and COX‐2, which promote melanocyte‐keratinocyte crosstalk [3, 4].

Tranexamic acid (TXA) has garnered significant attention in dermatological research for its multifaceted skin benefits, particularly its ability to inhibit the expression of inflammatory markers. TXA effectively inhibits melanocyte activity through modulation of the plasminogen‐plasmin system, consequently attenuating melanin synthesis. Numerous studies have demonstrated TXA's efficacy in improving conditions such as melasma, post‐inflammatory hyperpigmentation, and overall skin tone. Its anti‐inflammatory properties make TXA a promising ingredient in the development of cosmetic formulations aimed at achieving clearer, more radiant skin [4, 5, 6, 7, 8].

Mandelic acid (MA), an alpha hydroxy acid (AHA) derived from bitter almonds, is renowned for its gentle exfoliating properties. Unlike other AHAs, MA's larger molecular structure allows for slower penetration into the skin, making it suitable for sensitive skin types. MA's antibacterial activity against *Cutibacterium acnes* and *Staphylococcus epidermidis* arises from its capacity to lower skin pH below 4.0, creating an unfavorable environment for bacterial proliferation while promoting keratinocyte desquamation. Its anti‐inflammatory effects are mediated through inhibition of NF‐κB and IL‐6/COX‐2 pathways, reducing erythema and pustule formation in acne lesions. MA's ability to enhance skin renewal makes it a valuable ingredient in cosmetic formulations aimed at improving skin health and appearance [9, 10, 11, 12].

Skin permeability enhancement is a critical area of research in both pharmaceutical and cosmetic sciences, aiming to improve the delivery of active ingredients into the skin for enhanced efficacy. Various mechanisms have been explored to achieve this goal, with most relying on physical methods such as microneedling, iontophoresis, and sonophoresis. These techniques, while effective, often require specialized equipment and expertise, limiting their accessibility and widespread application [13, 14]. Chemical penetration enhancers, such as surfactants, alcohols, and fatty acids, have also demonstrated significant potential in improving skin permeability. However, their use is often accompanied by drawbacks, including skin irritation and barrier disruption, due to their potential to damage the skin's organic lipid structure [15, 16]. In contrast, ion‐pairing technology offers a promising alternative. Unlike physical methods or traditional chemical enhancers, ion‐pairing facilitates the formation of diverse combinations with substances capable of forming counterions or hydrogen bonds. This mechanism overcomes the limitations of previous techniques, retains the inherent properties of each substance, and is increasingly recognized as a significant advancement in the field of cosmetics. By leveraging the unique interactions between ions, ion‐pairing technologies can enhance skin permeability while minimizing potential side effects [17, 18, 19, 20].

This study focuses on the TXA‐MA complex. Dynamic light scattering (DLS), ^1^H NMR study, and Fourier‐transform infrared spectroscopy (FT‐IR) analyses confirmed the formation of the ion‐pairing complex. Subsequently, ex vivo and in vitro evaluations demonstrated that the TXA‐MA complex exhibited superior skin permeation and inhibition of inflammatory marker expression compared to TXA alone. Furthermore, in vivo human evaluations indicated enhanced efficacy of TXA‐MA complex in improving skin pigmentation.

By enhancing skin permeation and inhibiting inflammatory marker expression, the TXA‐MA complex holds significant potential as an effective cosmetic ingredient for improving skin pigmentation. This study aims to validate its benefits and propose its application in cosmetic formulations.

## 2 Materials and Methods

### 2.1 Formation of the TXA‐MA Complex

TXA (Sigma‐Aldrich, St. Louis, MI, USA) and MA (Sigma‐Aldrich, St. Louis, MI, USA) were prepared for the TXA‐MA ion‐pairing complex (TXA‐MA complex) in a molar ratio of 1:1 (TXA:MA). To synthesize the TXA‐MA complex, equimolar amounts of TXA and MA were combined to achieve a final concentration of 50% in deionized water. The mixture was vigorously stirred at 70°C for a duration of 20 to 30 min until a homogeneous solution was achieved. Subsequently, the solvent was evaporated using an appropriate process, resulting in the successful production of the TXA‐MA complex.

### 2.2 Structure Analysis of the TXA‐MA Complex

To confirm the formation of the TXA‐MA complex, the following evaluations were conducted. First, zeta‐potential measurement was investigated by using DLS with a Zetasizer Nano ZS instrument (Malvern Instruments, UK). Scattered light was detected at an angle of 173° using the non‐invasive back scattering (NIBS) technique. Triplicate samples were measured three times each at 25°C. The measurement was initiated within 120 s after sample preparation. Second, FT‐IR (Spectrum Two, Perkin Elmer) analysis revealed the occurrence of energy changes in the functional groups using the attenuated total reflectance (ATR) mode due to the intermolecular interaction between the TXA/MA simple mixture and the TXA‐MA complex. Through complementary interpretation of the data, the difference between the TXA‐MA complex and the TXA/MA simple mixture was elucidated, confirming the formation of the ion‐pairing complex. Finally, ^1^H NMR (Bruker, Avance III 600 MHz NMR spectrometer, UK) analysis was used to check the formation of the TXA‐MA complex. Deuteroxide (D_2_O) was used as a solvent in the ^1^H NMR study. Samples (TXA, MA, and the TXA‐MA complex) were dissolved in the solvent and analyzed.

### 2.3 In Vitro Skin Permeation Study

An in vitro permeation study was conducted using Franz diffusion cells. Porcine skin membranes measuring 2.5 cm × 2.5 cm × 1 mm were purchased (Micropig Franz Cell Membrane, Apures, Korea) and used for the in vitro skin permeation study. The receptor compartment of the Franz diffusion cell was filled with 3 mL of PBS buffer, and the porcine skin was placed in between. Subsequently, each prepared sample (based on a 3% concentration of TXA, including TXA alone, TXA/MA mixture, and TXA‐MA complex solution) was introduced into the donor compartment of the Franz diffusion cell in 100 µL aliquots and then applied uniformly to the upper surface of the porcine skin by rolling 10 times using the tip of a 3 mL Eppendorf tube (*n* = 2). The donor compartment was then taped, and each sample was placed in a temperature‐ and humidity‐controlled chamber at 37.5°C and 50% humidity for 1, 2, 4, and 6 h. After 1, 2, 4, and 6 h, respectively, the remaining sample from the donor compartment was wiped clean, and to quantify the amount of TXA penetrating the porcine skin, each sample was added to 1 mL of PBS buffer and homogenized using a Precellys 24 homogenizer (Bertin Technologies, Montigny, France). The resulting mixture was then centrifuged at 13 000 rpm for 10 min using a Centrifuge 5427R (Eppendorf, Germany) to separate the supernatant for analysis of TXA within the skin. The TXA analysis samples obtained from the permeated porcine skin were prepared by sampling the reservoir solution [21, 22].

HPLC (Agilent 1260 series) was used for the analysis of TXA content in the prepared samples, with the Agilent OPA (o‐Phtaladehyde) injection program used to derivatize TXA before analysis. The separation column used was a ZORBAX Eclipse AAA (4.6 m × 150 mm, 5 µm), with the eluent consisting of a mixture of A (acetonitrile, methanol, and DI water in a ratio of 45:45:10) and B (40 mM sodium dihydrogen phosphate [pH 7.8]) in a ratio of 0:100 for 2 min and 57:43 for 18 min for a gradient separation. The flow rate was set at 1.5 mL/min, and quantitative analysis was performed at 338 nm compared to a standard reagent. For the extraction of each sample, 2 g was taken into a 20 mL flask and diluted with 10 mL of 0.1 N HCl, followed by ultrasonic extraction and filtration.

### 2.4 Cell Culture and Treatment

Human epidermal keratinocyte cell lines (HaCaT) were grown in Dulbecco's modified Eagle's media (DMEM, Gibco, Waltham, MA, USA) containing 10% fetal bovine serum (FBS, Gibco, Waltham, MA, USA) at 37°C in a humidified CO_2_ incubator (95% air, 5% CO_2_). For each sample treatment, HaCaT cells were seeded onto 60‐mm culture plates at a density of 2–5 × 10^5^ cells/mL and cultured for 24 h. The growth medium was then removed, and the cells were exposed to UVB radiation (10 mJ/cm^2^). After UVB exposure, the cells were treated with appropriately diluted samples in FBS‐free DMEM and incubated for 24 h.

### 2.5 Real‐Time PCR Analysis

Total RNA was isolated from keratinocyte cells using the Easy Blue reagent, and cDNA synthesis was performed using the reverse transcriptase enzyme. Real‐time PCR was then carried out as the main step in the polymerase chain reaction. The primer sequences used for PCR are listed in Table 1. To calculate the extent of inhibition by the test substance on the increased expression of UVB‐induced inflammation‐related cytokines (IL‐1α, IL‐6, IL‐8, COX2), the following formula was applied:

$$\mathrm{Inhibition}\;\mathrm{rate}\;\;\left(\%\right)=1-\left[\left(\frac{\mathrm{Treated}-\mathrm{CTL}}{\mathrm{UVB}\;\mathrm{radiation}-\mathrm{CTL}}\right)\times 100\right]$$

where Treated: Gene expression fold change when substance is treated after UVB radiation; UVB radiation: Gene expression fold change under UVB radiation; and CTL: Gene expression fold change under non‐UVB radiation.

**TABLE 1:** List of primers for real‐time PCR.

Gene		Sequence
IL‐1α	Forward	5'‐AAGTGTTGACAGGCCGTATG
	Reverse	5'‐TACCAGACTTCGCTCCCTCT
IL‐8	Forward	5'‐ATGACTTCCAAGCTGGCCGT
	Reverse	5'‐TCCTTGGCAAAACTGCACCT
IL‐6	Forward	5'‐AAGCCAGAGCTGTGCAGATGAGTA
	Reverse	5'‐TGTCCTGCAGCCACTGGTTC
COX2	Forward	5‘‐CACTACATCCTGACCCACTT
	Reverse	5‘‐ATGCTCCTGCTTGAGTATGT
GAPDH	Forward	5'‐GTCAGTGGTGGACCTGACCT
	Reverse	5'‐AGGGGTCTACATGGCAACTG

### 2.6 Clinical Assessment of Skin Pigmentation

To evaluate the improvement in skin pigmentation, suitable participants were selected following the clinical evaluation criteria established by experts. The selection criteria were based on the ITA value, a skin color measurement indicator that follows the CIE Lab* color model. The test area was considered eligible if its ITA value was above 28 and the difference in ITA values between test areas was less than 5. According to these criteria, 32 female participants between the ages of 22 and 57 with healthy skin (mean age: 45.81 ± 7.91 years) and no sensitivity or hypersensitivity were evaluated. The study was conducted under the supervision of an observer and followed standard operating procedures. Prior to participation, informed consent was obtained from each participant (study period: October 8, 2024, to November 4, 2024; IRB approval number: IRB‐240919T001).

The clinical study consisted of four distinct groups, each receiving a different treatment for direct comparison:

- The Control group received no treatment.
- The Base group was treated with a base ampoule containing no active ingredients.
- The TXA group was treated with an ampoule containing TXA.
- The TXA‐MA group was treated with an ampoule containing the TXA‐MA complex.

For the selected participants, a designated area of 1 × 1 cm^2^ was marked on both ventral forearms, at a distance of 5 to 10 cm from the wrist, with a spacing of 3 cm. The minimum erythema dose (MEDu) for each individual was calculated using the ITA value measurements, and an artificial pigmentation was induced by exposing the test area to an irradiance equivalent to 1.5MED using a Multi‐port Solar Simulator 601‐300 W (Solar Light, USA). The participants then visited the testing facility, where baseline measurements (before product application) were taken on day 0. During the 4‐week experimental period, participants applied the test product to the designated area twice daily, in the morning and evening, gently spreading it. At Week 2 and Week 4, participants visited the testing facility again, and the skin brightness of the pigmented area was measured using the ANTERA 3D CS system (Miravex Limited, Ireland) to analyze the changes. Throughout the analysis period, any actions that could stimulate the forearms were prohibited, and participants were instructed to refrain from skincare practices such as massages, esthetic treatments, and the use of any cosmetic products, including peeling products. All participants waited for 30 min under controlled conditions (temperature 20°C–22°C, humidity 40%–60%) before the evaluation. The variable used for skin brightness evaluation was CIE L* (A.U.). The percentage of improvement in skin brightness was calculated using the following formula:

$$\mathrm{Improvement}\;\mathrm{in}\;\mathrm{skin}\;\mathrm{brightness}\;\;\left(\%\right)=\frac{\left(L-L_{0}\;\right)}{L_{0}}\;\;\times 100$$

where *L_0_*: Initial skin brightness (L); *L*: skin brightness after 2 and 4 weeks.

### 2.7 Statistics

All statistical analyses were performed using IBM SPSS Statistics 21.0 software (IBM, Armonk, NY). The results were expressed as the mean ± standard deviation (SD). Differences between time points or groups were analyzed using *t* tests with the statistical significance set at *p* < 0.05.

## 3 Results

### 3.1 Preparation and Characterization of the TXA‐MA Complex

To verify the successful formation of the TXA‐MA complex, zeta‐potential and FT‐IR analyses, which are commonly used to confirm ion‐pairing, were conducted. When an ion‐pairing complex forms, the influence of the surface charge of each substance decreases, resulting in a zeta‐potential value close to zero. To confirm this, 1% solutions of TXA, MA, a TXA/MA mixture, and the TXA‐MA complex were prepared, and their zeta potential was analyzed. As shown in Table 2, the results were as follows: TXA (−4.71 mV), MA (−28.4 mV), TXA/MA mixture (−10.86 mV), and TXA‐MA complex (−3.3 mV). The zeta potential measurements revealed that MA exhibited a stronger surface charge compared to TXA. Although the mixture showed a reduction in surface charge compared to MA alone, the decrease was not significant. In contrast, the TXA‐MA complex exhibited a zeta‐potential value close to zero, confirming the formation of the ion‐pairing structure.

**TABLE 2:** Zeta potential of TXA, MA, TXA/MA mixture, and TXA‐MA complex (1% solution).

	TXA	MA	TXA/MA mixture	TXA‐MA complex
Zeta potential	−4.71 mV	−28.4 mV	−10.86 mV	−3.3 mV

**FIGURE 1:**
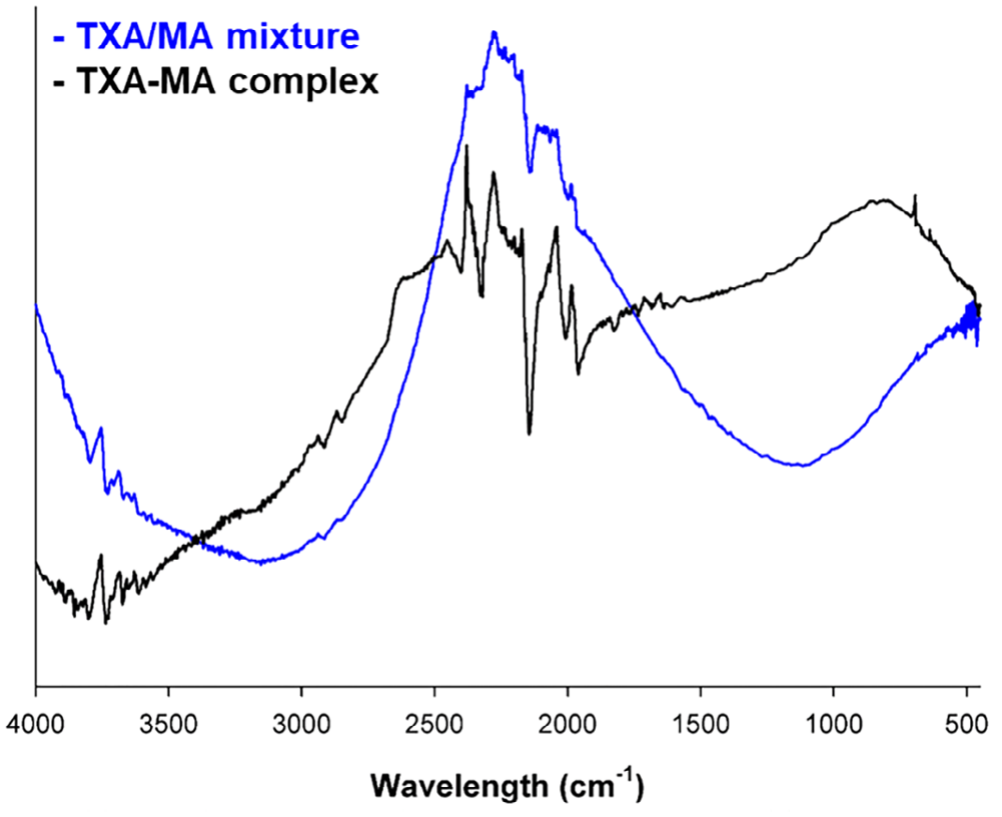
FT‐IR spectroscopic analysis of the TXA/MA mixture and the TXA‐MA complex.

FT‐IR analysis was utilized to verify the formation of ion‐pairing complexes by examining the energy changes between functional groups. Ion‐pairing complexes, formed via charge‐charge interactions or hydrogen bonding, are influenced by the intermolecular interactions between the substances, leading to shifts in IR peak positions. Figure 1 presents the FT‐IR spectra of the TXA/MA mixture and the TXA‐MA complex. Notably, the TXA‐MA complex exhibited peak changes at 2911, 2843, 2617 (O‐H stretching of carboxylic acid), and 1626 cm^−1^ (N‐H bend of amine group) compared to the TXA/MA mixture. These findings suggest that the formation of the TXA‐MA complex involves stronger intermolecular interactions between the two substances, distinguishing the complexes from simple mixtures.

**FIGURE 2:**
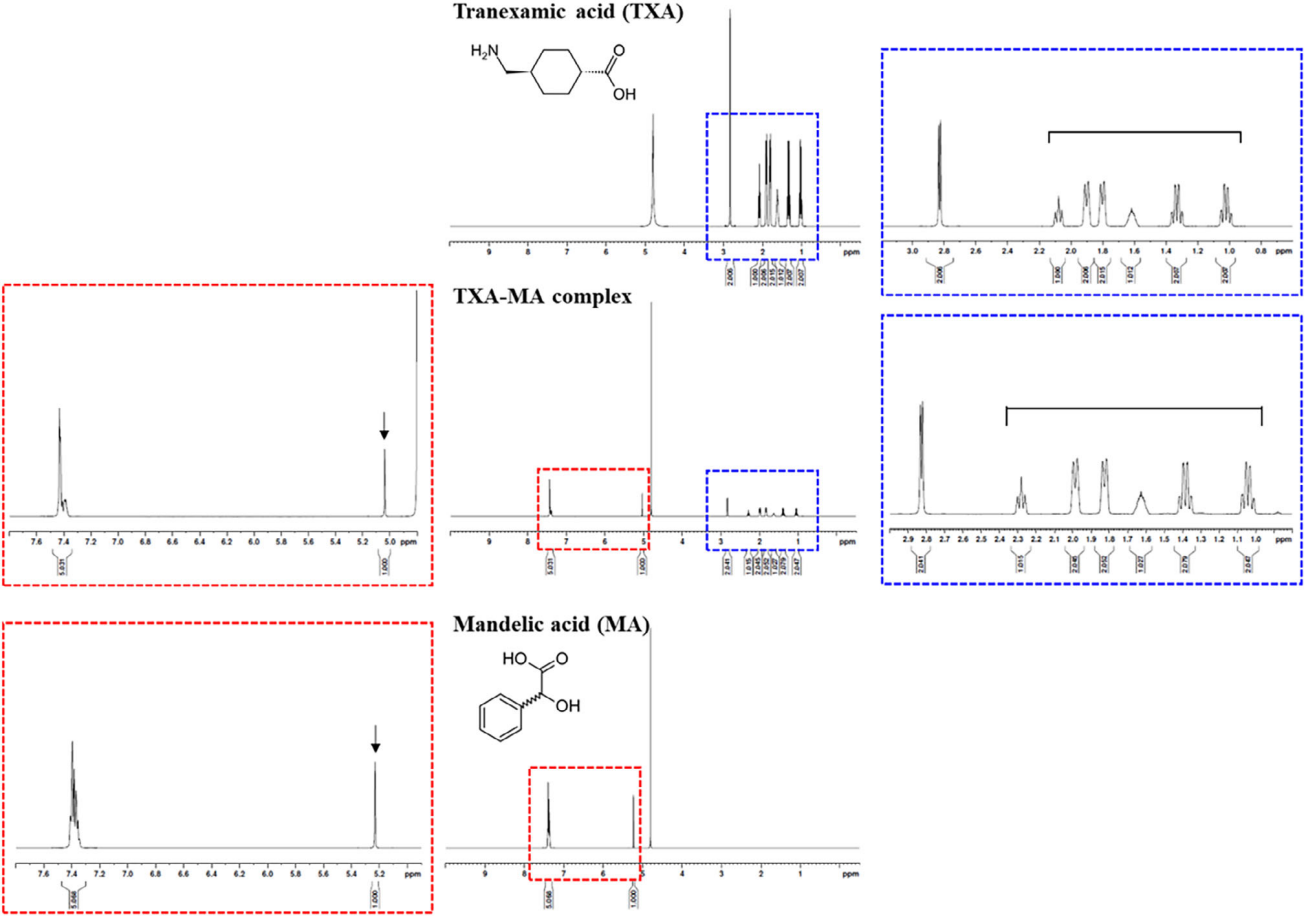
^1^H NMR spectra of TXA, MA, and TXA‐MA complex.

Comprehensive ^1^H NMR analyses of TXA, MA, and the TXA‐MA complex (Figure 2) elucidated the structural basis of their ion‐pair interaction. The cyclohexane methylene protons of TXA (δ 1.0–1.9 ppm range in free TXA) exhibited an upfield shift (δ 1.0–2.0 ppm range) in the complex, consistent with protonation of the amine group (‐NH_2_ → ‐NH_3_^+^). Concurrently, MA's methine proton (δ 5.23 ppm in free MA) shifted downfield to δ 5.03 ppm in the complex, reflecting electron density redistribution upon deprotonation of the carboxylic acid (‐COOH → ‐COO^−^). The multi‐technique validation (NMR, FT‐IR, and zeta potential) conclusively demonstrates that the TXA‐MA complex is stabilized by the ion‐pairing structure between TXA and MA [22, 23, 24, 25, 26].

### 3.2 In Vitro Skin Permeation Studies

An in vitro skin permeability study was conducted using the Franz diffusion cell technique to investigate the skin permeability of the TXA‐MA complex compared to TXA alone. Porcine skin, which shares morphological and functional similarities with human skin, was used as a reliable animal model for transdermal absorption assessment. After mounting the skin on the Franz diffusion cell, solutions of TXA and TXA‐MA complex were prepared at a concentration of 3% TXA. Each sample, 100 µL in volume, was applied to the porcine skin and evenly distributed by rolling.

**FIGURE 3:**
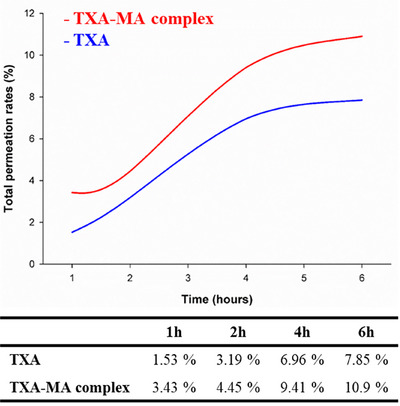
Permeation rates of TXA alone and the TXA‐MA complex via Franz diffusion cell using porcine skin at 1, 2, 4, and 6 h.

Following 1‐, 2‐, 4‐, and 6‐h permeation periods, the applied solutions were removed, and the amount of TXA permeated into the porcine skin and reservoir was measured, respectively. Permeation rates were calculated based on the initial amount of 100 µL of 3% TXA and the amount of TXA that permeated. Based on the calculated permeation rates for each time point, the permeation rate graphs for TXA and TXA‐MA complex are shown in Figure 3. It can be observed that TXA‐MA complex exhibits approximately 2.24 times faster permeation than TXA at 1 h and around 1.4 times faster permeation at 2, 4, and 6 h. As time progresses beyond 1 h, skin permeation increases at a similar rate, and the graph reflects a saturation trend, demonstrating a pattern comparable to the reported skin permeation results using porcine skin.

The enhanced skin permeation observed with the TXA‐MA complex can be attributed to various mechanisms, with the permeation enhancement effect due to ion pairing. First, ion pairing formation reduces the surface charge of particles to approach ‘0.’ Our zeta‐potential measurements confirmed a value close to neutrality (−3.3 mV), facilitating interactions with the lipid‐rich stratum corneum [22, 27].

### 3.3 Inhibitory Effect of the TXA‐MA Complex on Inflammatory Factor Expression

**FIGURE 4:**
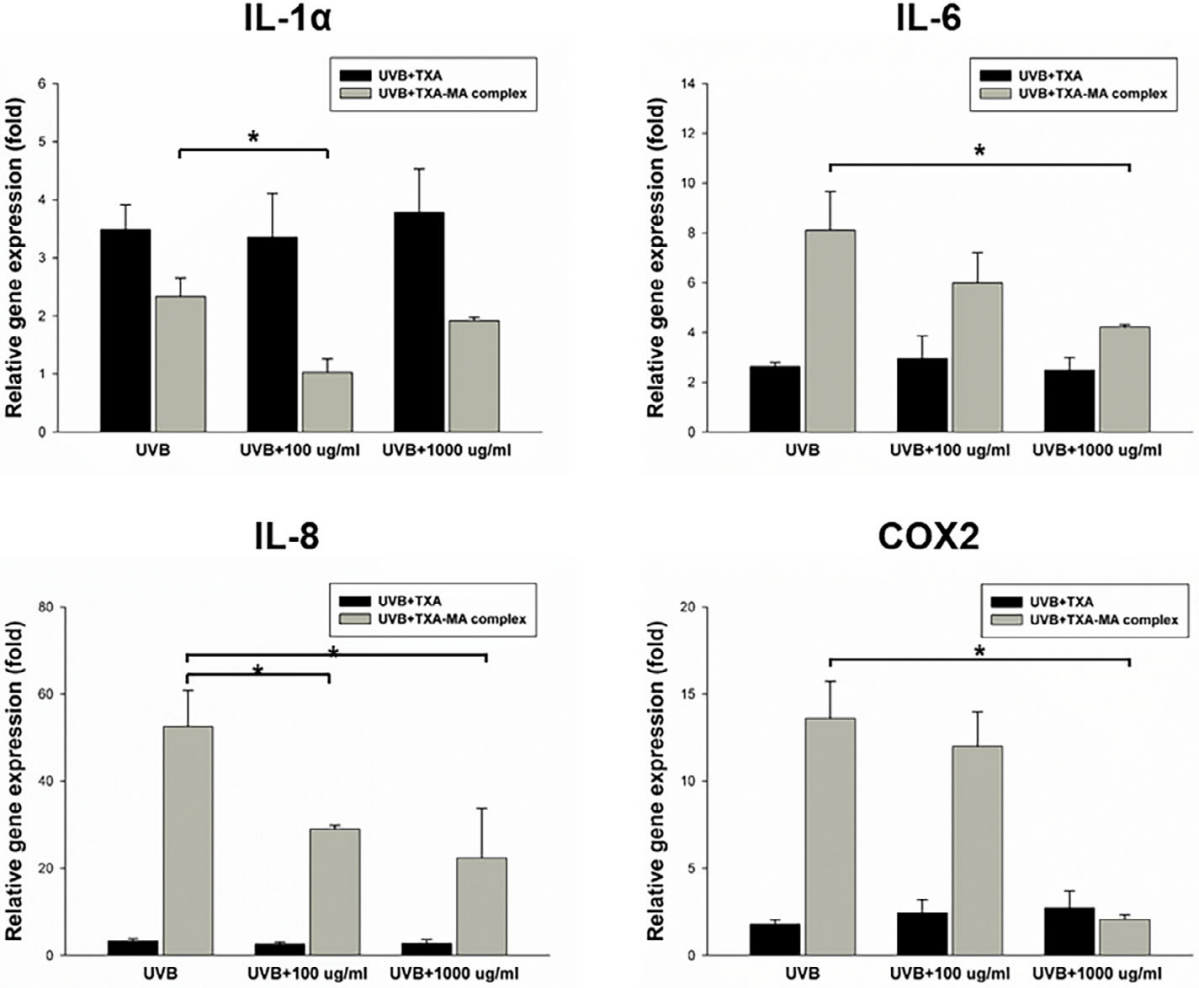
Relative gene expression (fold) of IL‐1α, IL‐6, IL‐8, and COX2. Data represent the means ± SD and comparison between UVB radiation sample and treated sample (*p < 0.05).

To investigate whether the TXA‐MA complex enhances the efficacy of improving skin pigmentation compared to TXA alone, we analyzed its impact on the expression changes of inflammation‐related genes IL‐1α, IL‐6, IL‐8, and COX2 induced by UVB (10 mJ/cm^2^) irradiation in human keratinocytes. The results of UVB treatment on keratinocytes showed an increase in the expression of IL‐1α, IL‐6, IL‐8, and COX2 compared to the untreated group, confirming the influence of UVB stimulation on actual inflammatory gene expression. Subsequently, we analyzed the extent of gene expression changes by applying TXA and TXA‐MA complex at concentrations of 100 and 1000 µg/mL, respectively. Figure 4 shows the analysis of relative gene expression for each inflammatory marker according to the application of UVB, UVB+TXA, and the UVB+TXA‐MA complex. As TXA is known to have anti‐inflammatory effects, most cases showed a decrease in inflammatory marker expression compared to the UVB‐treated group. When comparing inhibition rates using the equation described in the experimental methodology, it can be deduced that the TXA‐MA complex demonstrates a higher efficacy in suppressing the expression of inflammatory mediators, in comparison to TXA alone, in all tested scenarios. This confirms that the TXA‐MA complex has superior inhibitory effects on inflammatory marker expression compared to TXA alone, demonstrating its excellent efficacy as a skin pigmentation‐improving ingredient. The structural synergy of the TXA‐MA complex, driven by ion‐pairing interactions confirmed through FT‐IR and zeta‐potential analyses, enables concurrent modulation of the plasminogen signaling pathway and oxidative stress. This synergy is further amplified by enhanced skin permeability, which potentiates the complex's ability to suppress cytokine expression through improved epidermal bioavailability.

### 3.4 Effect of TXA‐MA complex on Improvement of Skin Pigmentation Staining as Clinical Assessment

To assess the efficacy of the TXA‐MA complex in improving pigmentation in actual skin, a clinical evaluation was conducted. Thirty‐two participants were exposed to artificial pigmentation on the inner forearms using an artificial UV emitter. Subsequently, for a duration of 4 weeks, the participants applied four different formulations twice daily, in the morning and evening, to the same locations: a control (no treatment), a base ampoule without active ingredients, a TXA‐applied ampoule, and a TXA‐MA complex‐applied ampoule. The change in skin brightness corresponding to the improvement in pigmentation was analyzed using the ANTERA 3D CS device. As shown in Table 3, after 2 weeks, the control group exhibited 5.02%, the base ampoule showed 5.394%, TXA revealed 5.376%, and the TXA‐MA complex demonstrated 5.794% improvement. Following the 4 weeks, the control group exhibited 6.092%, the base ampoule showed 6.188%, TXA revealed 6.564%, and the TXA‐MA complex demonstrated 6.746% improvement. Assuming the recovery rate of the control group represents natural recovery, the ratios compared to the control group indicate the improvement effect on pigmentation. Therefore, after 2 weeks of usage, the TXA‐MA complex exhibited 15.42%, TXA revealed 7.09%, and the base ampoule showed 7.45% improvement in pigmentation. After 4 weeks, the TXA‐MA complex exhibited 10.75%, TXA revealed 7.75%, and the base ampoule showed 1.58% improvement in pigmentation. In both the 2‐week and 4‐week results, the TXA‐MA complex‐applied ampoule demonstrated a significantly higher improvement effect on pigmentation compared to the base ampoule and the TXA‐applied ampoule (*p* < 0.001). Thus, it can be concluded that the TXA‐MA complex enhances the pigmentation improvement effect of TXA.

**TABLE 3:** Improvement rates of skin brightness (L* value) over a period of 2 and 4 weeks based on different ampoule applications (*n* = 32).

	Control	Base ampoule	TXA applied ampoule	TXA‐MA complex applied ampoule
2 week	5.020%***	5.394%***	5.376%***	5.794%***
4 week	6.092%***	6.188%***	6.564%***	6.746%***

*Note*: The table presents a comparison between the 0‐, 2‐, and 4‐week results for each evaluation (*p < 0.05, **p < 0.01, ***p < 0.001).

## 4 Conclusions

The TXA‐MA complex is formed through ion‐pairing between TXA and MA, and the complex formation was confirmed through zeta‐potential and FT‐IR analyses. In vitro evaluation confirmed the enhanced skin permeability expected through ion‐pairing formation, as well as the reinforced anti‐inflammatory effect of TXA in suppressing inflammatory responses. Application of the complex on human skin demonstrated improved reduction of pigmentation, indicating that the TXA‐MA complex exhibits higher efficacy in treating pigmentation than TXA alone. These results highlight the potential of TXA‐MA complex as a new ingredient for tone correction and anti‐aging cosmetics. This study has several limitations and areas for improvement. Most notably, for cosmetic applications, it is essential to conduct comprehensive clinical evaluations addressing a wide range of skin phenomena. To provide more meaningful information for real cosmetic use, it will be necessary to assess the effects of various formulation types and their broader impacts on different skin functions.

## Conflicts of Interest

The authors declare no conflicts of interest.

## Data Availability

The data used to support the findings of this study are included within the article or available from the corresponding author upon request.
